# Sinonasal diffuse large B-cell lymphoma in a patient with Wiskott–Aldrich syndrome: A case report and literature review

**DOI:** 10.3389/fimmu.2022.1062261

**Published:** 2023-01-12

**Authors:** Xiwen Sun, Chunyu Luo, Ru Tang, Song Mao, Ying Zhu, Chonghui Fei, Mengyu Wang, Shaolin Tan, Shiyao Zhang, Jiayao Zhou, Hai Lin, Zhipeng Li, Weitian Zhang

**Affiliations:** ^1^ Shanghai Key Laboratory of Sleep Disordered Breathing, Department of Otolaryngology-Head and Neck Surgery, Otolaryngology Institute of Shanghai JiaoTong University, Shanghai Sixth People’s Hospital Affiliated to Shanghai Jiao Tong University School of Medicine, Shanghai, China; ^2^ Department of Neonatology, Children’s Hospital of Soochow University, Suzhou, China; ^3^ Department of Ophthalmology, Shanghai Sixth People’s Hospital Affiliated to Shanghai Jiao Tong University School of Medicine, Shanghai, China; ^4^ Jinzhou Medical University Postgraduate Training Base (Department of Otolaryngology-Head and Neck Surgery, Shanghai Sixth People’s Hospital), Shanghai, China

**Keywords:** Wiskott-Aldrich syndrome, diffuse large B cell lymphoma, sinonasal, surgery, case report

## Abstract

Wiskott–Aldrich syndrome (WAS) is a rare primary immunodeficiency disease with a predisposition towards autoimmunity and lymphoproliferative diseases. Non-Hodgkin lymphoma (NHL) is reported to be the predominant form of malignant tumor in WAS sufferers. Diffuse large B-cell lymphoma (DLBCL) is one of the most common types of NHL while it is uncommon to occur in paranasal sinuses and especially when associated with WAS. In this article, we report a unique case of WAS associated with DLBCL in paranasal sinuses and review the major publications of WAS-related lymphomas that occurred in the head and neck area. This study extends the available therapies for WAS-related lymphomas and emphasizes the significance of recognition for sinonasal lymphomas in WAS patients presenting with sinusitis.

## 1 Introduction

Wiskott–Aldrich syndrome (WAS) is a rare X-linked disorder affecting 1 in 50,000 and 1 in 250,000 male newborns with disease-causing gene mapped to Xp11.22-p11.23 (1). The gene encodes WAS protein (WASP), whose mutations are associated with combined immunodeficiency, platelet defect, eczema, and increased susceptibility to autoimmune disorders and cancers (2). At the early stage, overwhelming infections and serious bleeding were the leading causes of WAS-related death (3). Afterward, with the modern prophylactic and treatment modalities that have led to a decreased incidence of lethal infections and bleeding, the major life-threatening complications in WAS-affected children became autoimmune disorders and malignant tumors (3, 4). Diffuse large B-cell lymphoma (DLBCL) is one of the most common types of non-Hodgkin lymphoma (NHL) (5). It is reported that NHL is the predominant form of malignancy in patients with WAS while DLBCL is rare, especially in paranasal sinuses (4, 6). Herein, we report a unique case of WAS with sinonasal DLBCL, which had extensively invaded the skull base and caused visual impairment. His medical procedures are reviewed in detail, and the clinical presentation, mechanism, treatment, outcomes, and diagnosis of this type of disease are discussed. Sinonasal malignancy from WAS should be evaluated in patients presenting with sinusitis symptoms. Prompt recognition may avoid unnecessary surgical intervention. However, optic nerve sheath fenestration (ONSF) is urgent when the tumor has caused visual loss. Timely recognition and treatment offer the best prognosis for recovery.

## 2 Case presentation

**Figure 1:**
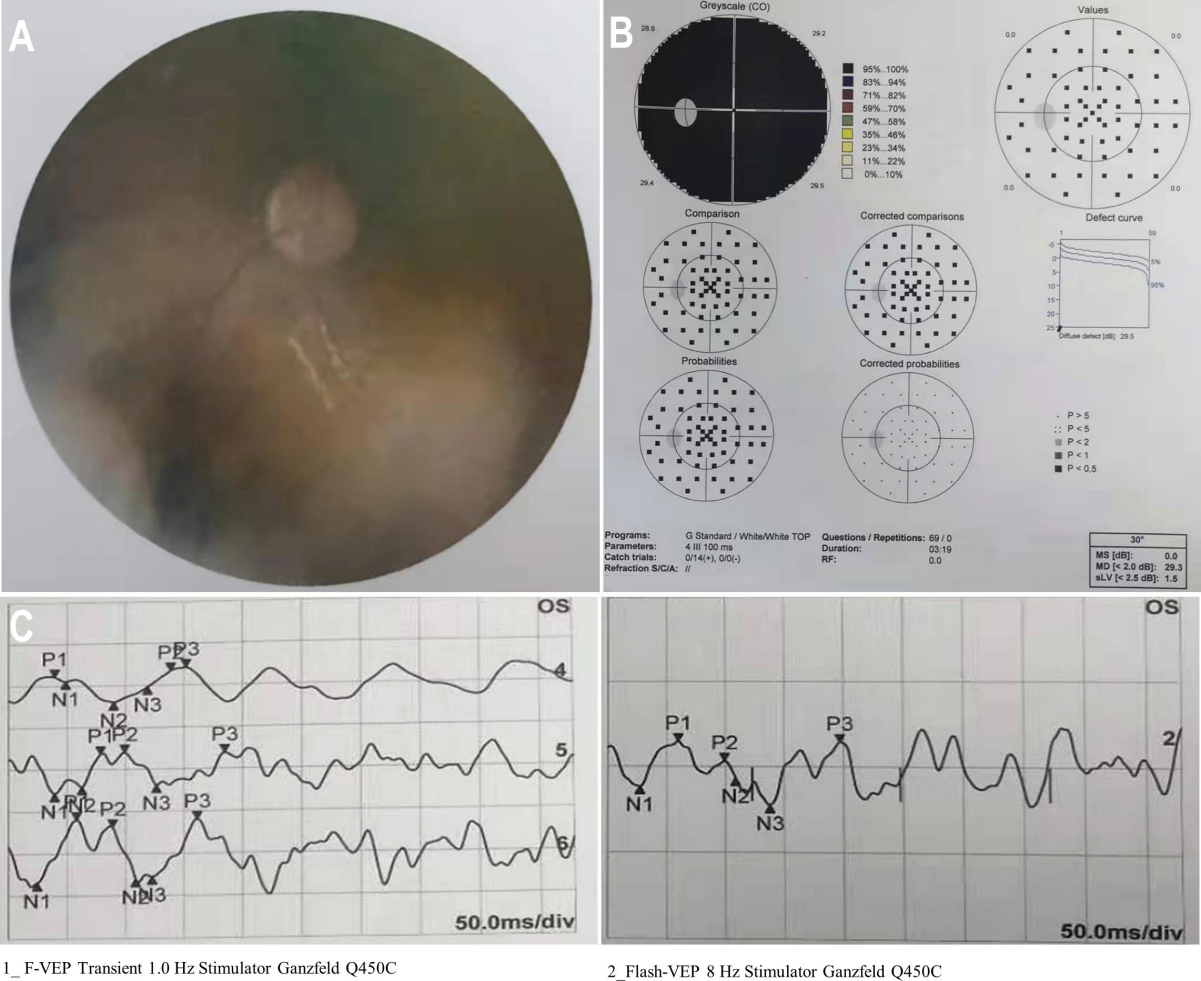
Preoperative condition of the left eye. **(A)** A funduscopic examination of the left eye revealed a hazy fundus. **(B)** Automated perimetry showed total visual field defect in the left eye. **(C)** F-VEPs of the left eye showed a reduced amplitude.

**Figure 2:**
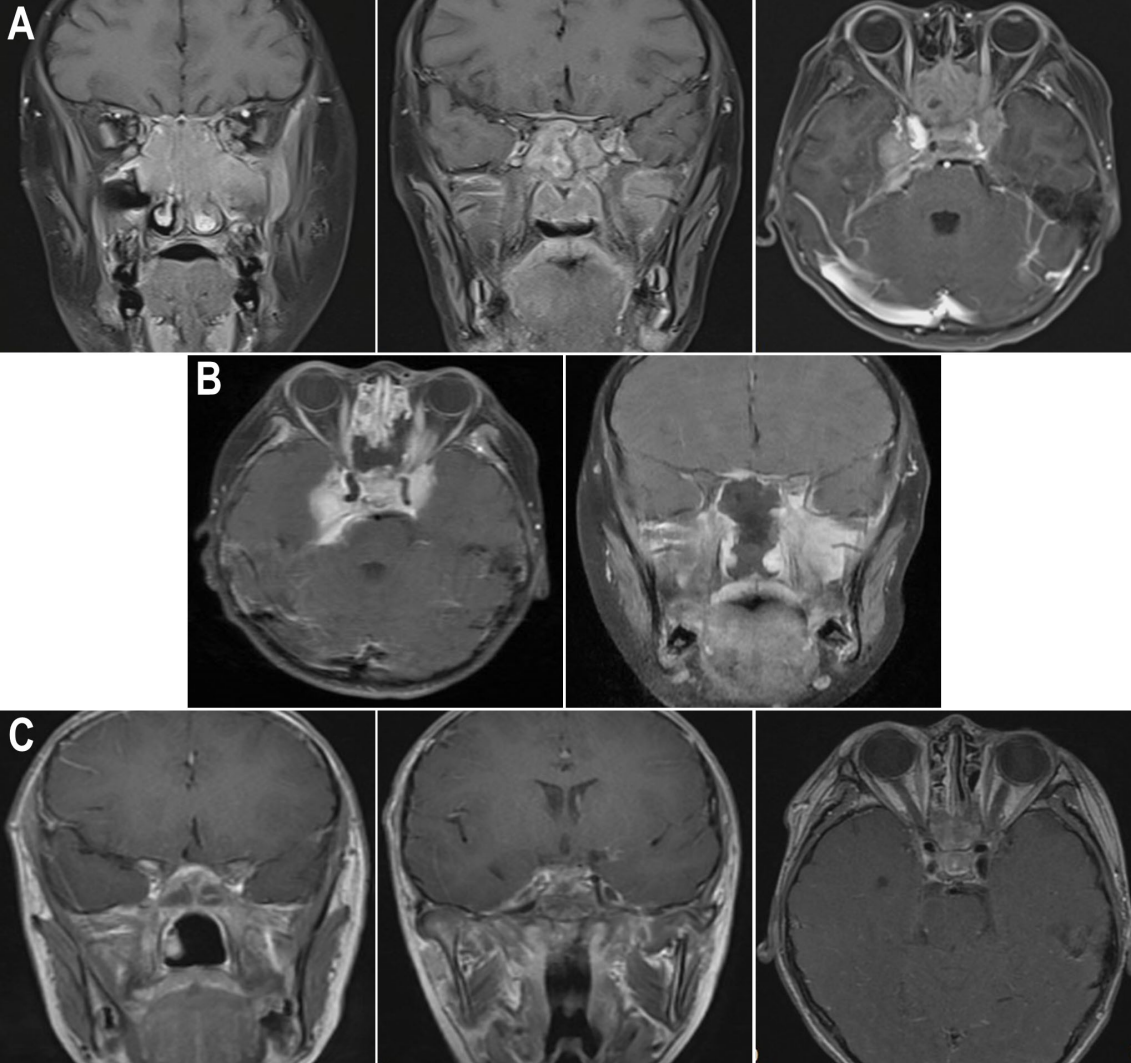
Enhanced nuclear MRI images of the patient during different periods. **(A)** Pre-op sagittal MRI images showed that the mass invaded the nasal cavity, ethmoid sinus, and sphenoid sinus, and compressed the optic nerve; pre-op axial MRI image revealed that the mass invaded the middle and posterior cranial fossa. **(B)** Post-op MRI images showed that intraoperative decompression of the optic nerve was performed. **(C)** Post-op MRI images showed the condition of the optic nerve and intracranial tumor after 1 month of HSCT.

**Figure 3:**
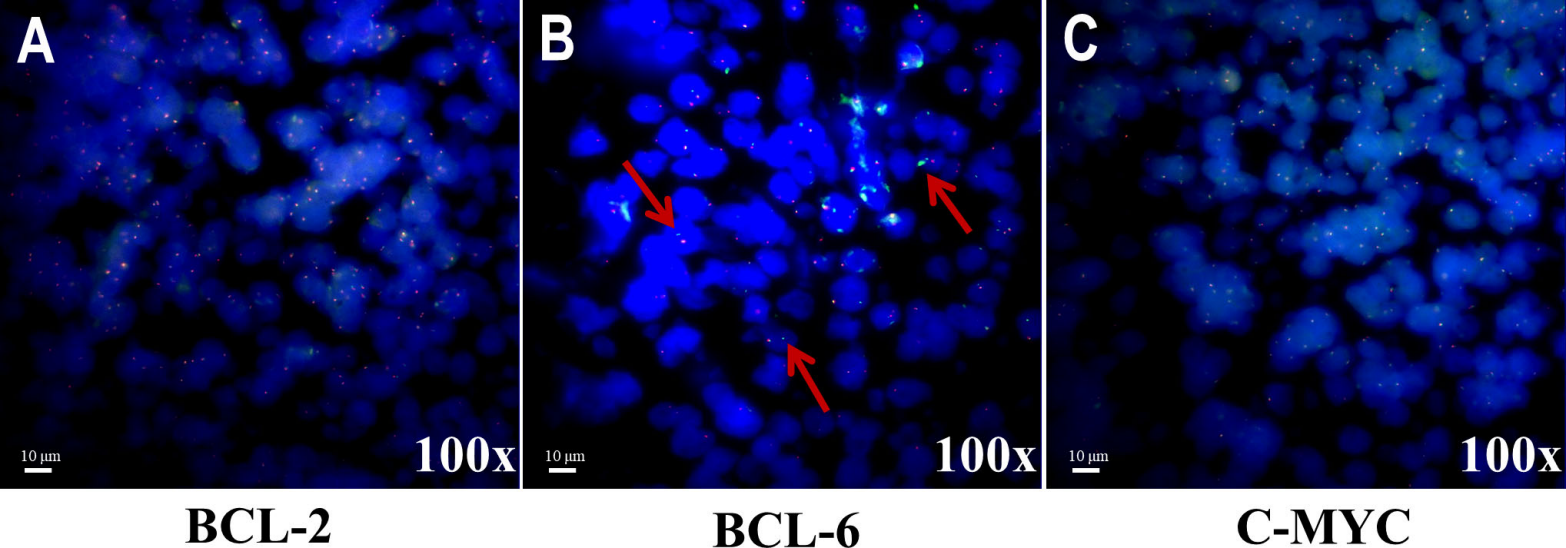
Representative images of translocation statuses by FISH analysis for three genes.**(A)** Tumor nuclei were negative for translocation of BCL-2. **(B)** Tumor nuclei were positive for translocation of BCL-6. Multiple tumor nuclei were seen with distinct red and green signals (highlighted by the red arrows). **(C)** Tumor nuclei were negative for translocation of C-MYC.

**Figure 4:**
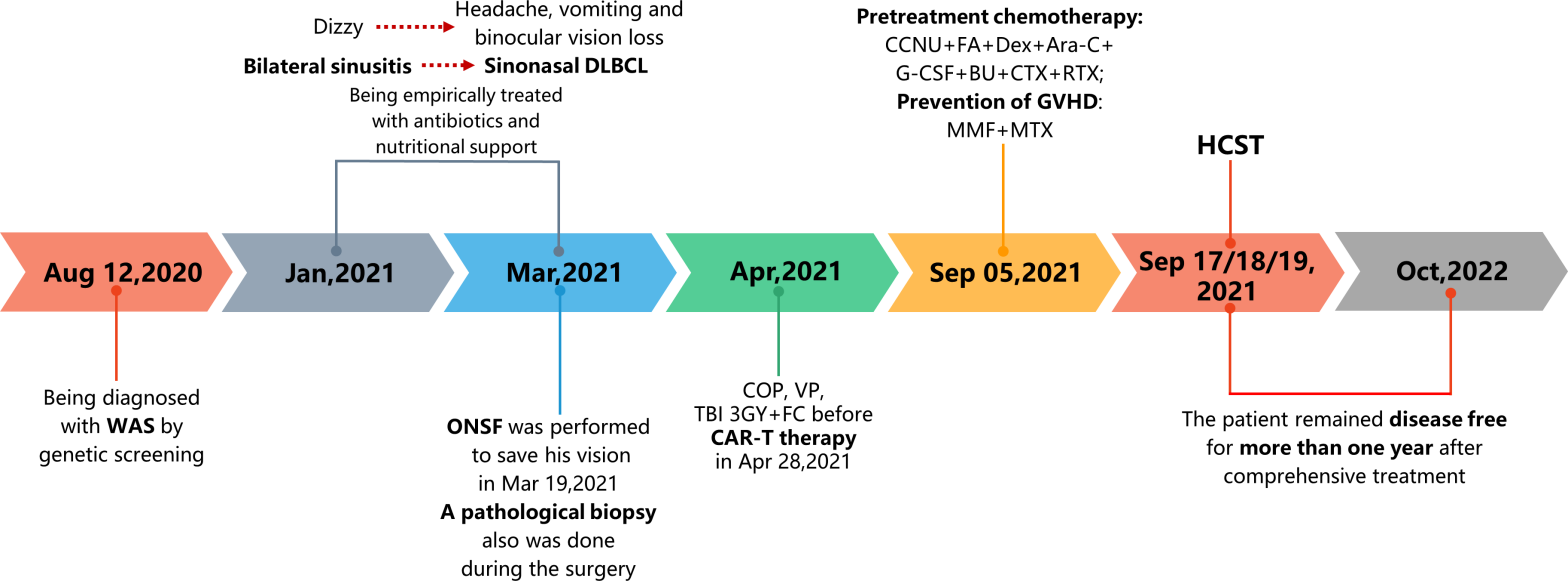
Timeline scheme of the major clinical events of the patient.WAS, Wiskott–Aldrich syndrome; ONSF, optic nerve sheath fenestration; COP, cyclophosphamide, vincristine, and prednisolone; VP, vinorelbine and prednisone; TBI, total body irradiation; FC, fluorouracil and carboplatin; CAR-T therapy, chimeric antigen receptor T-cell immunotherapy; CCNU, lomustine; FA, fludarabine; Dex, dexamethasone; Ara-C, cytosine arabinoside; G-CSF, granulocyte colony-stimulating factor; BU, busulfan; CTX, cyclophosphamide; RTX, rituximab; GVHD, graft-versus-host disease; MMF, mycophenolate mofetil; MTX, methotrexate; HSCT, hematopoietic stem-cell transplantation.

A 12-year-old boy diagnosed with WAS was admitted to our hospital in March 2021 with a 3-month history of dizziness, headache, vision loss in both eyes, and intermittent vomiting. In January 2021, the boy felt dizzy and undertook a head CT scan that showed bilateral sinusitis. Then, he was empirically treated with antibiotics and nutritional support. Around March 2021, the boy developed a headache, vomiting, and binocular vision loss. His vital signs were normal when doing a physical examination on admission. However, the vision of his right eye was 0.5 and the left eye had only light perception. Testing of cranial nerves (CNs) revealed that the boy had left abducens nerve paresis and right lower group CN total paresis. The funduscopic examination, perimetry, and F-VEPs showed damage to his left optic nerve (**Figure 1**). Enhanced MRI showed irregular mass had invaded the nasal cavity, ethmoid sinus, sphenoid sinus, and middle and posterior cranial fossa and compressed the bilateral optic canals, in bilateral paranasal sinuses and nasopharynx areas. The lateral wall of the sinus was destructed. The mass presented with diffuse and slightly longer T1-weighted imaging (T1WI)/T2-weighted imaging (T2WI), slight hyperintensity on fat-suppressed imaging, and significant enhancement. On imaging, the diagnosis was highly likely lymphoma (**Figure 2A**). The dura mater, cranium, posterior fossa of the trigeminal nerve root, bilateral cavernous sinuses, and internal carotid arteries were eroded to varying degrees. The patient’s medical history was remarkable for thrombocytopenia and recurrent eczema, and he was initially suspected to have WAS shortly after his birth, but it was not until August 2020 that he was diagnosed with WAS through genetic screening. Soon after, ONSF was performed to save his vision. Intraoperatively, the ethmoid tumor was removed to expose the orbital cardboard. The sphenoid plateau was invaded and reduced from the midline to both sides. The optic nerve canal was further completely exposed, and the tumor around the optic canal was removed to decompress the optic nerve (**Figure 2B**). The final immunohistochemistry findings showed that the malignant cells were positive for CD10, IRF4/MUM1, BCL-6, CD20, and CD79α, and negative for CD3, TIA-1, CD207/langerin, and CD5. The Ki67 was 80% positive. Genetic testing of the malignant cells was positive for BCL-6-related gene translocation but negative for BCL-2- and C-MYC-related genes, and the gene translocations were detected by fluorescence *in situ* hybridization (FISH) (**Figure 3**). These results confirmed the diagnosis of DLBCL with germinal center B type. A few days after the surgery, the boy was transferred to the Department of Hematology for further treatment. According to recent follow-up records, the boy underwent chemotherapy and chimeric antigen receptor (CAR) T-cell immunotherapy before the bone marrow transplant. Before chemotherapy and CAR-T therapy, the patient had a low T-cell count (<700/μl) but a compensatory increase in NK cell count (>600/μl), which were resolved after the following treatment. However, no abnormalities were detected in the levels of cytokines including IL-2, IL-4, IL-6, IL-10, TNF-α, IFN-γ, and IL-17A in the patient’s peripheral blood. The condition regimens patient used for CAR-T therapy and allogeneic HSCT are shown in **Figure 4**. The patient’s response to each therapy was unremarkable, expect on the fifth day of CAR-T therapy, the patient developed red rash on the trunk and limbs, pleural effusion, and decreased blood pressure, which was considered to be related to the cytokine release syndrome induced by allogeneic CAR-T therapy. Tocilizumab and dexamethasone were given, and ulinastatin was added to improve capillary permeability, and norepinephrine was used to increase blood pressure. There was no further deterioration of vision in both eyes after the surgery (**Figure 2C**) and he remained disease free for 1 year after comprehensive treatment. On the basis of radiographic evidence of tumor shrinkage and the absence of distant metastasis, we considered this patient to be in “partial remission.” The main treatment steps for the patient are shown in **Figure 4**.

## 3 Discussion

WAS is a serious X-linked recessive primary immunodeficiency disorder characterized by eczema, thrombocytopenia, immune deficiency, and susceptibility to autoimmune diseases or malignancies which was named by the two physicians who described it, Alfred Wiskott (1937) and Robert Aldrich (1954) (1, 7). The boy we mentioned in this report had a single T deletion in exon 10 of the WAS gene (c.1032delT), which led to a frameshift in the Val codon at position 345 (p.Val345fs) (**Table 1**). Mutations in the WAS gene have various effects on the level of WASP, which, in turn, correlates with the diversity of the disease. Except for classic WAS, the WAS variant also leads to X-linked thrombocytopenia (XLT) or X-linked neutropenia (XLN) which reflect the different clinical phenotypes and clinical outcomes of WAS (3, 7).

**Table 1:** Genetic variant strongly associated with disease.

Gene name	Chromosome localization	Transcript localization	Nucleotide change	Amino acid change	Mutation property	Zygotic property
WAS	chrX:48547148	Exon10	c.1032delT	p.Val345fs	Frame shift	Homozygote

The possibility of cancer occurrence in patients with WAS is a significant concern. Approximately 13% of patients with WAS would develop malignant tumors, and the average age at diagnosis is 9.5 years (5). The most frequent malignant tumor is lymphoma, predominantly non-Hodgkin type presenting in extranodal sites induced by EBV infection (5, 7, 8). DLBCL is the most common pathologic type of NHL, which is also the most frequently encountered variant in WAS-affected patients (4). Unfortunately, dozens of cases are poorly characterized concerning the limited molecular detection techniques at early times (9). We located sporadic case reports of extranodal DLBCL in WAS in the published literature, with only one instance occurring in the brain (5), one in the pharyngeal (4), one in the orbit (10), one in the larynx (11), and two cases occurring in the systemic extranodal organs (12, 13) (**Table 2**). Therefore, the involvement of the nasal cavity and paranasal sinuses is an atypical but probable tumor site presentation that has not been previously reported.

**Table 2:** Extranodal DLBCL in WAS patients.

Study (year)	Type of study	Country	Staging of DLBCL	EBV status	Sex/Age	Presentation	Treatment	Outcome
Du S et al. (5) (2011)	Case report	American	DLBCL Ann Arbor IAE	Positive	14 years, male	Brain lesions	Chemotherapy and Rituxan	Died with active disease
Coccia P et all (4). (2012)	Case report	Italy	DLBCL Ann Arbor IAE	Negative	15 years, male	Epipharyngeal growth	Chemotherapy-LNH 97 with Rituximab at 75% dose	Alive and in remission at 3 years of last follow-up
Gilson D et all (10). (1999)	Case report	Britain	DLBCL Ann Arbor IAE	Negative	16 years, male	Orbital mass	Chemotherapy presentation-UKCCSG and radiotherapy	Alive till 10 years of last follow-up
Palenzuela G et all (11). (2003)	Case report	France	DLBCL Ann Arbor IAE	Positive	15 years, male	Acute laryngitis: laryngeal growth	CVP followed by local RT followed by laser debulking	Died with acute respiratory failure related to a lung infection
Nakanishi M et all (13). (1993)	Case report	Japan	DLBCL Ann Arbor IVB	Positive	20 years, male	Multiple CNS lesions and systemic extranodal organs	Local RT followed by acyclovir and adenosine arabinoside intravenously	Died with *Pseudomonas* sepsis
Yoshida K et all (12). (1997)	Case report	Japan	DLBCL Ann Arbor IVA	Positive	21 months, male	Systemic lymph nodes and extranodal organs	N/A	Died with active disease

CVP, Cyclophosphamide Oncovin Prednisolone; Chemotherapy-LNH 97, The Italian Association of Pediatric Hematology and Oncology LNH 97 clinical protocol; Chemotherapy Presentation-UKCCSG, A United Kingdom Children’s Cancer Study Group (UKCCSG) regimen; N/A, not available.

Despite the close relationship between WAS and hematolymphoid malignancies, the etiology of lymphoma in patients with WAS is not fully understood (4, 5, 14, 15). Although WASP is considered the main causative factor due to its effect on a variety of cell types and the multiple intracellular and extracellular events that followed, EBV infection may also provide a mechanistic explanation for lymphomagenesis in the majority of WAS-associated lymphoproliferative diseases (7, 12–14, 16). Immunosurveillance by T cells and NK cells, which is generally defective in classical WAS patients, may explain the pathogenesis of DLBCL in WAS patients. Patients with WAS are characterized by defective thymic output and an increased frequency of apoptosis, which may explain the common phenomenon of T-cell lymphopenia in WAS patients. In addition, mutations in the gene encoding WASp cause defects in filamentous actin (F-actin) branching, resulting in the inability of T cells to migrate to secondary lymphoid organs, form stable immune synapses, proliferate, and secrete cytokines (17). WASp-deficient NK cells are unable to rearrange their F-actin cytoskeleton, which is required for NK cell immunological synapse formation and NK cell cytotoxicity (18). Concerns regarding the number and function of T cells and NK cells in patients during treatment are of crucial importance, because they could promptly respond to the treatment effect of the patients, but the monitoring of cytokine levels in peripheral blood of patients often cannot reflect the situation in time, and it may be necessary to conduct multiple tests or use other precise methods to evaluate the functional status of T cells and NK cells. In the case we reported, the patient was EBV-negative. It is more likely that the primary immune defect in WAS increases susceptibility to lymphoma development by several pathways. However, the specific causes of DLBCL in WAS are still unclear.

The prognosis for lymphoma in WAS patients is still dismal due to their immunodeficiency (15). Allogeneic hematopoietic stem-cell transplantation (HSCT) remains the most curative therapy for WAS, with hematopoietic stem-cell (HSC) gene therapy and gene editing developing as optimistic treatment options (19). The most common treatment for DLBCL in WAS is still chemotherapy (4, 15). Surgery is an alternative option when the tumor involved critical blood vessels and nerves or the tumor blocked the respiratory and digestive tract. In this case, endoscopic sinus surgery was performed for his vision salvage and headache relief. Moreover, a pathological biopsy could also be done during the operation, which could determine the nature of the tumor for further treatment strategies. Minimally invasive surgery *via* nasal endoscopy is more suitable for patients with bleeding tendencies and basic diseases than open surgery.

The sinonasal malignancies initially cause very few symptoms. Moreover, due to the infrequently involved location, tumors in this area are often misdiagnosed as sinusitis at the early stage (20). As a result, such as the case we reported, a high index of suspicion of sinus malignancy in WAS patients with a history of chronic sinusitis in combination with cranial neuralgia or orbital symptoms and signs should be considered. Improved awareness of these symptoms and subsequent early diagnosis are important for the effective treatment of this rare and polymorphic disease.

## Data availability statement

The raw data supporting the conclusions of this article will be made available by the authors, without undue reservation.

## Ethics statement

The studies involving human participants were reviewed and approved by Ethics Committee of Shanghai Sixth People’s Hospital. Written informed consent to participate in this study was provided by the participants’ legal guardian/next of kin.

## Author contributions

WZ, ZL, RT, and XS conceived and designed the study. XS, CL, ZL, SM, ST, CF, and MW were in charge of the data collection and analysis. XS, CL, ZL, WZ, YZ, SZ, JZ, and HL wrote, reviewed, and/or revised the manuscript. All authors contributed to the article and approved the submitted version.
